# Texture analysis of MR images of patients with Mild Traumatic Brain Injury

**DOI:** 10.1186/1471-2342-10-8

**Published:** 2010-05-12

**Authors:** Kirsi K Holli, Lara Harrison, Prasun Dastidar, Minna Wäljas, Suvi Liimatainen, Tiina Luukkaala, Juha Öhman, Seppo Soimakallio, Hannu Eskola

**Affiliations:** 1 Medical Imaging Center, Tampere University Hospital, Tampere, Finland; 2 Department of Biomedical Engineering, Tampere University of Technology, Tampere, Finland; 3 Tampere Medical School, University of Tampere, Tampere, Finland; 4 Department of Neurosciences and Rehabilitation, Tampere University Hospital, Tampere, Finland; 5 Department of Neurosurgery, Tampere University Hospital, Tampere, Finland; 6 Science Center, Pirkanmaa Hospital District, Tampere, Finland; 7 Tampere School of Public Health, University of Tampere, Tampere, Finland; 8 Department of Emergency Medicine Acuta, Tampere University Hospital, Tampere, Finland

## Abstract

**Background:**

Our objective was to study the effect of trauma on texture features in cerebral tissue in mild traumatic brain injury (MTBI). Our hypothesis was that a mild trauma may cause microstructural changes, which are not necessarily perceptible by visual inspection but could be detected with texture analysis (TA).

**Methods:**

We imaged 42 MTBI patients by using 1.5 T MRI within three weeks of onset of trauma. TA was performed on the area of mesencephalon, cerebral white matter at the levels of mesencephalon, corona radiata and centrum semiovale and in different segments of corpus callosum (CC) which have been found to be sensitive to damage. The same procedure was carried out on a control group of ten healthy volunteers. Patients' TA data was compared with the TA results of the control group comparing the amount of statistically significantly differing TA parameters between the left and right sides of the cerebral tissue and comparing the most discriminative parameters.

**Results:**

There were statistically significant differences especially in several co-occurrence and run-length matrix based parameters between left and right side in the area of mesencephalon, in cerebral white matter at the level of corona radiata and in the segments of CC in patients. Considerably less difference was observed in the healthy controls.

**Conclusions:**

TA revealed significant changes in texture parameters of cerebral tissue between hemispheres and CC segments in TBI patients. TA may serve as a novel additional tool for detecting the conventionally invisible changes in cerebral tissue in MTBI and help the clinicians to make an early diagnosis.

## Background

Mild traumatic brain injury (MTBI) accounts for 70 - 90% of all treated brain injuries [1]. MTBI is usually caused by a relatively mild blow to the brain that causes just enough physical injury to possibly compromise the normal brain functions of memory, attention, mental organization, and logical thinking may be compromised. Damage to the brain is often found in the corpus callosum, brain stem, and in subcortical white matter (WM) regions at the site of impact or on the contralateral side after MTBI [2].

One of the biggest challenges in addressing neuropsychological functioning and recovery from MTBI is the diagnosing itself. A variety of neuroimaging modalities can be used to assist in making the diagnosis of MTBI [3], but currently CT scan and MRI are the modalities of choice as a diagnostic tool for acute MTBI. The vast majority of MTBI patients have normal CT scans, and although MRI has been found to be more sensitive to traumatic lesions than CT, most symptomatic patients also have normal MRI scans.

MR images of tissues contain a lot of microscopic information that may not be assessed visually and texture analysis (TA) technique provides the means for obtaining this information [4]. Texture is the visual cue due to the repetition of image patterns that can be described for example, as smooth or rough, regular or irregular, coarse or fine. Some textures display complex patterns but may appear visually regular and are therefore relatively easy to extract even by visual inspection. However, for textures that exhibit random appearance patterns where textural primitives are randomly placed it becomes much more difficult to recognize and interpreter these textures. These kind of random patterns rather than regular textures are more often encountered in medical images. Basically texture is an image feature which corresponds to both brightness value and pixel locations from which TA allows one to calculate mathematical patterns, texture features that can be used to discriminate and characterize the properties of tissues.

TA of MR images is a quantitative method that can be used to quantify and detect structural abnormalities in different tissues. TA can be divided into categories such as structural, model-based, statistical and transform, according to the means employed to evaluate the inter-relationships of the pixels [5]. Statistical methods are the most widely used in medical images. The statistical approaches analyze the spatial distribution of grey values, computing local features at each point in the image, and deriving a set of statistics from the distributions of the local features. Local features are defined by the combination of intensities at specific position relative to each point in image. Statistics are classified as a first-, second- or higher-order statistics according to the number of points which define the local feature. In first- order statistics image properties depend solely on individual pixel values, whereas second-order statistics are properties of pixel pairs [4]. First order statistics include mean grey scale, standard deviation of the mean, skewness (deviation of the pixel distribution) and the kurtosis (stepness of the pixel distribution) which can usually be detected visually. Second order statistically methods utilizes grey-level run-length measures and grey-level co-occurrence matrix. Methods based on second-order statistics tend to obtain higher discrimination indexes and can not be visually detected. Therefore the interest in medical image TA mainly lays in the random textures of second- or higher order. The most popular texture method for MR images seems to be the grey-level co-occurrence matrix first proposed by Haralick [6].

Many promising studies have been reported with TA in the classification of pathological tissues from normal tissues for example from the liver, breast, tumours with variable locations such as lymphomas and muscles [7-13]. With regard to TA of brain, texture parameters based on the histogram, co-occurrence matrix, gradient and run-length matrix have been shown to be good for the characterization of healthy and pathological human cerebral tissues [14-18]. Co-occurrence matrix-based TA has also been found to be sensitive in differentiating Alzheimer's disease patients from normal controls [19] and histologically proven hippocampal sclerosis (HS) from normal hippocampal cerebral tissue [20]. Mahmoud-Ghoneim et al. [21] have proposed a three-dimensional (3D) approach using co-occurrence matrix analysis to increase the sensitivity and specificity of brain tumor characterization and treatment follow-up with promising results. Ganeshan [22] and associates 3D selective- and relative-scale texture analysis to quantify the presence of grey-matter and white-matter textural abnormalities associated with schizophrenia concluding that 3D TA of brain MR enables detection of subtle distributed morphological features associated with schizophrenia. Kovalev and associates [23] also tested 3D co-occurrence matrix TA in analyzing cerebral tissue and glioma in T1-weighted MR-images. TA has also been used in analyzing age-related changes [24] and gender-related differences [25] with promising results.

In this study we concentrated on evaluating the ability of two-dimensional (2D) MRI-based TA to characterize the changes caused by MTBI in cerebral tissue by applying TA methods. To the best of our knowledge, there are no published studies on the application of quantitative MRI TA in studying MTBI.

## Methods

Patients with MTBI (GCS score 13-15) were recruited from the emergency room of Tampere University Hospital during the period 2006-2007. For the TA study 42 consecutive patients (17 male, 25 female; mean age ± SD, 38.8 ± 13.6 years; range 18 to 60 years) were included. Clinical examination on admission and CT examination on the day of the accident and MRI within three weeks from the day of admission were conducted on all patients. All patients met the criteria of MTBI according to the World Health Organization Collaborating Centre for Neurotrauma Task Force on Mild Traumatic Brain Injury [26]. Exclusion criteria for this study were age under 18 or over 65, severe traumatic brain injury, previous brain trauma, other major cognitive disorder, history of major alcohol or drug abuse. Ten healthy age and gender matched controls (4 males, 6 females; mean age ± SD, 39.8 ± 12.9 years; range 28 to 61 years) were also recruited to form a control group. All patients and healthy controls gave their written consent and the study was approved by the Ethics Committee of Tampere University Hospital. All 42 patients were evaluated to have a normal CT and MRI scan by a specialized radiologist. The patient's degree of consciousness was assessed to determine the severity of brain injury using the Glasgow Coma Scale (GCS) [27]. Possible loss of consciousness (LOC) was recorded (length in minutes or hours) as well as post-traumatic amnesia (PTA) (length in minutes or hours). A number of neurocognitive tests were also performed within 6 weeks of the injury.

### **MRI examinations**

All 42 patients were studied on a 1.5 Tesla MRI machine (Magnetom Avanto, Siemens Medical Solutions, Erlangen, Germany). The MRI machine is under quality control program, which includes daily, monthly, and quarterly measurements. Main magnetic field homogeneity and RF -amplifier properties are measured and controlled four times a year. A prescan normalisation filter was used for the correction of intensity inhomogeneity in images. The data used for homogenisation were acquired through a preliminary low-resolution measurement. An elliptical filter was used within the slice planes to improve the signal-to-noise ratio. The sequences included in the MRI protocol are presented in Table 1.

**Table 1:** Sequences included in the MRI protocol for MTBI patients.

Sequence	TR	TE	TI	Slice/gap	matrix	FOV	Flip angle
sagittal T1w 3D magnetization prepared gradient echo	1910	3.1	1100	1.0/0	256 × 256	250	15
axial T2w Turbo Spin Echo	44860	96	0	5.0/1.5	293 × 448	230	
axial FLAIR	9000	109	2500	5.0/1.5	256 × 256	230	
axial T2*w HEMO	800	26	0	7.0/2.0	256 × 256	230	20
axial SE EPI 3 scan diff (b = 0, b = 500, b = 1000)	3400	89	0	5.0/1.5	192 × 192	230	
sagittal FLAIR	8860	116	2500	2.0/2.0	256 × 256	230	
axial SE MDDW 12dir (b = 0, b = 1000)	3600	96	0	5.0/1.5	128 × 128	230	
axial SWI 3D	15	49	40	2.0/0	177 × 256	230	15

TR = repetition time

TE = echo time

TI = inversion time

FOV = field of view

### Texture analysis

For texture analysis an axial FLAIR (T2w FLAIR) and sagittal T1w 3D magnetization prepared gradient echo (T1w MPR) image series were selected from the whole MRI study. Three image slices from imaging sequences T2w FLAIR on three selected levels of interest and one slice from sequence T1w MPR were chosen for further analysis. Level 1 was level of mesencephalon, level 2 corona radiata and level 3 centrum semiovale. Level 4 was corpus callosum from sagittal view in caudo-cranial direction from the T1w MPR sequence. Image selection was performed with a DICOM viewer Osiris (Windows version 4.19, The Digital Imaging Unit (UIN) of the Service for Medical Computing (SIM) of the Radiology Department of the University Hospital of Geneva, Switzerland).

**Figure 1:**
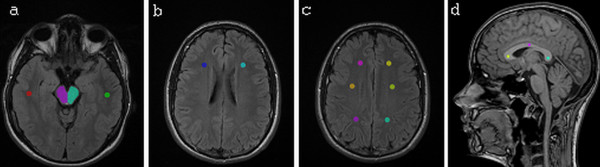
**Regions of interest drawn on the each level of interest**. Figure 1a: an axial FLAIR (T2w FLAIR) image in level 1. Figure 1b: an axial FLAIR (T2w FLAIR) image in level 2. Figure 1c: an axial FLAIR (T2w FLAIR) image in level 3. Figure 1d: sagittal T1w 3D magnetization prepared gradient echo (T1w MPR) in level 4. Regions of interest (ROIs) are drawn in the images.

TA was performed with the software package MaZda (MaZda 4.5, Technical University of Lodz, Institute of Electronics [28]) specially designed for texture analysis by Materka and co-workers as part of the European COST B11 and the following COST B 21 programs. For each MR image regions of interest (ROI) were manually placed symmetrically on the left and right hemispheres on each level of interest. For level 1 ROIs were drawn by hand in the area of mesencephalon (ROI size around 1200 pixels depending on the size of the mesencephalon), both left and right side. Circular ROIs (177 pixels) were placed both sides in WM (Figure 1a). For level 2 circular ROIs were placed both sides in WM (177 pixels) (Figure 1b). For level 3 three circular ROIs (177 pixels) were placed in both sides in WM from anterior to posterior (Figure 1c). Circular ROIs (68 pixels) were also placed on the splenium, body and rostrum of the corpus callosum (Figure 1d). The ROIs were carefully placed so they did not overlap any microhemorraghes, macroscopic hemosiderin deposits or hyperintensities, which were observed in few patients. The ROI drawing was done manually by person with special interest in developing quantitative radiology methods in clinical use.

The comparison of texture features was made between the left and right sides and between segments of CC to ascertain any changes in texture parameters between hemispheres or segments on patients and on controls.

After determining the ROIs we calculated texture features based on image histogram, the co-occurrence matrix, the run-length matrix, the absolute gradient and the autoregressive model and wavelets [28]. Run length matrix parameters were calculated in four directions: horizontal (0°), vertical (90°), 45° and 135° and co-occurrence matrix parameters were calculated in five distances (1, 2, 3, 4 and 5 pixels), four times for each distance (in directions *θ *= 0°, 45°, 90° and 135°). All of these texture features (See Additional file 1) were calculated for each ROI.

The grey level normalization of each ROI was performed using a method which normalizes image intensities in the range [μ-3σ, μ+3σ]. This method has been shown to give the best results in MRI texture classification among different normalization methods [29]. This was done to minimize the influence of contrast variation and brightness. To determine 10 texture features with the highest discriminative power for separation and classification we used feature selection method Fisher coefficient (F) provided by MaZda [28]. The Fisher criterion usually produces a set of features with a high discriminatory potential which are also highly correlated with each other. The top 10 feature selections were made when comparing texture features between hemispheres of WM in different levels and features in the area of mesencephalon and features between segments of CC.

### Data analysis

Statistical analyses were run for every texture feature. Differences in texture features between hemispheres in different tissues (the right vs. the left side of mesencephalon and WM) were analyzed by Wilcoxon Signed Ranks. The parameters in WM between three different levels and WM anterior-posterior (front, middle and back) on level 3, in the same hemisphere, were tested with the Friedman test. Texture parameters calculated from the segments of CC (rostrum, body and splenium) were also analyzed with the Friedman test. Similar tests were performed on the group of healthy controls. These analyses were performed using SPSS for Windows, version 14.0.2. (SPSS Inc., Illinois, USA). A p-value of under 0.05 was considered statistically significant.

## Results

### **Analyses of mesencephalon**

We tested all raw texture parameters to find out how many and which of the 277 parameters differed statistically between hemispheres. The number of texture parameters (n = 277) which were statistically significantly different (*p *< 0.05) analyzed with Wilcoxon test in the area of mesencephalon between hemispheres is presented in Table 2.

**Table 2:** Numbers of parameters having statistically significant differences (*p *< 0.05) between hemispheres analyzed with Wilcoxon test.

	Mesencephalon	
Texture parameter groups	Patients	Controls
Histogram (n = 11)	7	3
GrM (n = 5)	1	0
COM (n = 220)	90	30
RLM (n = 20)	4	0
ARM (n = 5)	3	2
Wavelet (n = 16)	2	2
**Total (n = 277)**	**107**	**37**

The total number of evaluated texture parameters is 277. The total number of parameters having statistically significant differences between hemispheres in the area of mesencephalon is in bold face.

GrM: gradient matrix; COM: co-occurrence matrix; RLM: run-length matrix; ARM: autoregressive model; n: number parameters in each group.

The parameters which differed statistically significantly were mainly based on the co-occurrence matrix. The patients had clearly more differences in texture features between hemispheres than the healthy controls. The healthy controls had no significantly differing run-length matrix based parameters unlike the patients.

The ten most discriminative texture features for separation of hemispheres in the area of mesencephalon as identified by calculation of Fisher coefficients, were mainly derived from the co-occurrence matrix in both patients and controls. The p-values for the most discriminative texture parameters on patients and on controls selected with the Fisher method are shown in Table 3.

**Table 3:** The ten most discriminating parameters according to the Fisher (*F*-) coefficient and corresponding Wilcoxon test p-values.

Mesencephalon					
**Most Discriminative Texture Parameters on Patients**	**p-values (patients)**	**p-values (controls)**	**Most Discriminative Texture Parameters on Controls**	**p-values (controls)**	**p-values (patients)**

Teta4	<0.001*	0.105	S(4,-4)DifVarnc	0.002*	0.519
Teta3	<0.001*	0.020*	S(5,-5DifVarnc	0.004*	0.465
S(5,5)Entropy	<0.001*	0.105	Teta3	0.020*	<0.001*
S(5,5)AngScMom	<0.001*	0.020*	S(5,-5)DifEntrp	0.004*	0.413
S(4,4)AngScMom	<0.001*	0.432	Teta2	0.014*	<0.001*
S(4,4)Entropy	<0.001*	0.322	S(4,-4)DifEnrtp	0.002*	0.372
Teta2	<0.001*	0.014*	S(3,-3)DifVarnc	0.002*	0.160
S(1,-1)DifVarnc	<0.001*	0.375	WavEnLL_s-2	0.027*	0,472
S(1,1)DifVarnc	<0.001*	0.375	S(5,-5)Contrast	0.002*	0.833
S(3,3)AngScMom	<0.001*	0.492	S(4,-4)Contrast	0.002*	0.432

The ten most discriminating parameters according to the Fisher (*F*-) coefficient and their corresponding Wilcoxon test p-values are calculated between hemispheres in the area of mesencephalon for both patients and controls.

Teta, vectors of autoregressive model; AngScMom, angular second moment; SumAverg, sum average; DifEntrp, difference entropy; DiffVarnc, difference variance; WavEnLL, energy of wavelet coefficients in subband LL.

* p-values of under 0.05 are considered statistically significant

Especially features derived from autoregressive model; Teta2, Teta3 and Teta4 (p < 0.001) were significantly different between hemispheres in patients and also in controls. Other parameters selected with the Fisher coefficient consisted mainly of parameters derived from the co-occurrence matrix. These were statistically different in patients but not in controls and vice versa.

### Analyses of white matter

Again we tested all raw texture parameters to find out how many and which of the 277 parameters differed statistically between hemispheres in WM in different levels of interest. The number of texture parameters (n = 277) which were statistically significantly different (*p *< 0.05) analysed with Wilcoxon test in WM between hemispheres in patients and healthy controls are set out in Table 4.

**Table 4:** Numbers of parameters having statistically significant differences (*p *< 0.05) between hemispheres analyzed with Wilcoxon test.

	White matter					
	WM Level 1		WM Level 2		WM Level 3	
Texture parameter groups	Patients	Controls	Patients	Controls	Patients	Controls
Histogram (n = 11)	8	9	9	9	8	6
GrM (n = 5)	0	0	0	0	0	0
COM (n = 220)	19	2	49	3	9	4
RLM (n = 20)	0	0	4	0	1	0
ARM (n = 5)	0	0	0	1	0	0
Wavelet (n = 16)	1	1	1	0	1	0
**Total (n = 277)**	**28**	**12**	**63**	**13**	**19**	**10**

The total number of evaluated texture parameters is 277. The total number of parameters having statistically significant differences between hemispheres in the cerebral white matter is in bold face.

GrM: gradient matrix; COM: co-occurrence matrix; RLM: run-length matrix; ARM: autoregressive model; n: number parameters in each group.

In the level of corona radiata (level 2) there were clearly more significantly different parameters between hemispheres than in other levels in patients. In level 2 there were also clearly fewer texture differences in controls than in patients.

The ten most discriminative texture features for separation of WM in the left and right hemispheres varied clearly between the three levels. The features were mainly histogram-based or derived from the co-occurrence matrix. The p-values for the most discriminative texture parameters in patients and in controls selected with the Fisher method in level 2 are shown in Table 5.

**Table 5:** The ten most discriminating parameters according to the Fisher (*F*-) coefficient and corresponding Wilcoxon test p-values.

White matter					
**Most Discriminative Texture Parameters on Patients**	**p-values (patients)**	**p-values (controls)**	**Most Discriminative Texture Parameters on Controls**	**p-values (controls)**	**p-values (patients)**

**LEVEL 2**					
S(4,4)Correlat	<0.001*	0.492	S(5,5)SumVarnc	0.002*	0.008*
S(4,4)Contrast	0.001*	0.557	S(5,5)Correlat	0.004*	0.008*
S(4,4)SumVarnc	<0.001*	0.695	S(4,4)InvDfMom	0.064	0.003*
S(4,4)InvDfMom	0.003*	0.064	WavEnLH_s-3	0.922	0.359
S(4,4)DifVarnc	0.002*	0.770	S(5,5)Contrast	0.010*	0.015*
S(5,5)Correlat	0.008*	0.004*	S(0,1)SumAverg	0.164	0.114
S(2,-2)AngScMom	<0.001*	0.396	S(1,-1)SumAverg	0.105	0.225
S(0,3)DifEntrp	0.002*	0.695	S(5,-5)SumVarnc	0.105	0.253
S(5,5)Contrast	0.015*	0.010*	S(5,-5)Correlat	0.105	0.603
S(5,5)SumVarnc	0.008*	0.002*	Teta1	0.064	0.274

The ten most discriminating parameters according to the Fisher (*F*-) coefficient and their corresponding Wilcoxon test p-values are calculated between hemispheres in the cerebral white matter in the level of corona radiata (level 2) for both patients and controls.

Correlat, correlation; DiffVarnc, difference variance; AngScMom, angular second moment; Teta, vectors of autoregressive model; SumAverg, sum average; InvDfMom, inverse difference moment; SumVarnc, sum variance; DifEntrp, difference entropy; WavEnLH, energy of wavelet coefficients in subband LH.

* p-values of under 0.05 are considered statistically significant

The most discriminative texture parameters in WM on patients and on controls varied between levels and between patients and controls. Only a few parameters were significantly different between hemispheres in both patients and controls.

The texture parameters of WM between different levels were analyzed with the Friedman test in order to find out whether the texture differed in the same hemisphere between levels. It was observed that many of the texture parameters of WM on level 1 were statistically significantly different from parameters on levels 2 and 3. Texture parameters in the same hemisphere of WM anterior-posterior (front, middle and back) on level 3 were also analyzed and it was observed that the texture parameters in the posterior region differed from the anterior and central regions in both hemispheres.

### Analyses of the corpus callosum

We tested all raw texture parameters to find out how many and which of the 277 parameters differed statistically between segments of CC. The number of texture parameters (n = 277) which were statistically significantly different (*p *< 0.05) analyzed with Friedman test in the segments of CC is presented in Table 6.

**Table 6:** Numbers of parameters having statistically significant differences (*p *< 0.05) between segments of CC analyzed with Friedman test.

Corpus callosum						
	patients			controls		
Texture parameter groups	Rostrum	Body	Splenium	Rostrum	Body	Splenium
Histogram (n = 11)	3	6	1	1	1	0
GrM (n = 5)	0	0	0	0	0	0
COM (n = 220)	3	33	4	4	1	3
RLM (n = 20)	0	0	0	0	0	0
ARM (n = 5)	0	1	0	0	0	0
Wavelet (n = 16)	0	9	0	1	2	0
**Total (n = 277)**	**6**	**49**	**5**	**6**	**4**	**3**

The total number of evaluated texture parameters is 277. The total number of parameters having statistically significant differences between the segments of corpus callosum is in bold face.

GrM: gradient matrix; COM: co-occurrence matrix; RLM: run-length matrix; ARM: autoregressive model; n: number parameters in each group.

In the segments of CC the body of CC had statistically significantly differing from the other segments on patients. In healthy controls there were clearly fewer significantly different parameters.

The ten most discriminative texture features for the separation of segments of CC as identified by calculation of Fisher coefficients, were mainly derived from the co-occurrence matrix and wavelet based features in both patients and controls. The p-values for the most discriminative texture parameters in patients and in controls selected with the Fisher method are shown in Table 7.

**Table 7:** The ten most discriminating parameters according to the Fisher (*F*-) coefficient and corresponding Wilcoxon test p-values.

Corpus callosum					
Most Discriminative Texture Parameters on Patients	p-values (patients)	p-values (controls)	Most Discriminative Texture Parameters on Controls	p-values (controls)	p-values (patients)
WavEnLL_s-2	<0.001*	0.001*	WavEnLL_s-2	0.001*	<0.001*
WavEnLH_s-2	<0.001*	0.006*	WavEnLH_s-2	0.006*	<0.001*
S(5,0)SumOfSqs	0.001*	0.601	S(4,-4)SumAverg	0.030*	0.220
S(3,0)Contrast	<0.001*	0.368	S(5,-5)SumVarnc	0.007*	0.699
S(4,0)SumOfSqs	0.003*	0.316	S(2,-2)SumEntrp	0.710	0.847
S(4,0)Contrast	<0.001*	0.368	S(1,-1)SumOfSqs	0.368	0.110
S(1,0)DifVarnc	<0.001*	0.368	S(5,-5)SumAverg	0.012*	0.190
S(2,0)Contrast	<0.001*	0.316	S(0,1)SumOfSqs	0.135	0.073
S(1,0)Contrast	<0.001*	0.436	S(2,-2)SumOfSqs	0.368	0.404
S(0,1)SumVarnc	0.019*	0.436	S(1,0)SumOfSqs	0.046*	0.272

The ten most discriminating parameters, according to the Fisher (*F*-) coefficient and their corresponding Wilcoxon test p-values are calculated between segments of the corpus callosum for both patients and controls.

SumAverg, sum average; Sum Varnc, sum variance, SumEntrp, sum entropy; DiffVarnc, difference variance, SumOfSqs, sum of squares; WavEnLH, energy of wavelet coefficients in subband LH; WavEnLL, energy of wavelet coefficients in subband LL.

* p-values of under 0.05 are considered statistically significant

The only texture features statistically significant in both patients and controls were two wavelet based features, otherwise the statistically significantly differing features differed between these two groups.

## Discussion

The use of imaging to examine patients with MTBI has been investigated by a number of studies, and imaging abnormalities in CT, MRI and SPECT have all been associated with poor outcome on all modalities [30-33]. Although the imaging modalities have been developing fast in recent years, with many improvements especially in MRI techniques, such as diffusion-weighted MRI, DTI and new MRI sequences [34-36] it is still difficult to detect damaged lesions and make the diagnosis of MTBI on the basis of imaging findings. Some prior studies have demonstrated exclusive abnormalities on DWI, ADC, or DTI without overt structural damage seen in other sequences such as T1, T2 [34,37] The use of advanced imaging modalities [31,38,39] and different computer assisted detection (CAD) systems such as TA, which provides quantitative means of characterizing the properties of tissues in cases which tissue changes cannot be detected by direct inspection of the image may offer possible approaches on improving the prognostic capabilities of conventionally used MRI sequences.

We chose the MR images of MTBI patients for our study with the objective of detecting textural differences in different regions of cerebral tissue between the hemispheres. The purpose was to test the performance of TA to differentiate cerebral hemispheres and to characterize the changes caused by MTBI in cerebral tissue. Our study showed that there are significant differences in texture parameters in cerebral tissue between the hemispheres in MTBI patients and also differences between patients and healthy controls. We found texture differences between sides in the area of mesencephalon and between the hemispheres in WM, especially in the level of corona radiata and between different segments of CC. To the best of our knowledge there are so far no other studies of texture analysis of MTBI patients for comparison.

It has been established that MR images contain tissue-specific texture features which can be extracted by mathematical methods. It has been proven that TA can be used for classifying healthy and pathologic human cerebral tissue [14-16] and also distinguish different cerebral tissues. TA has also been used for distinguishing MS lesions from normal appearing - and normal white matter [40]. In light of our study we concur that TA can discriminate between different cerebral tissues and that different structures can also be distinguished from brain MR images. Traumatic brain injury is followed by activation of numerous proinflammatory mediators and glial cells. Both experimental and clinical data show activation of proinflammatory cytokines at the site of injury [41,42]. This together with an assumption of axonopathic changes in DTI might suggest inflammatory etiology of TA [43].

In our statistical tests on the raw parameters there were over a hundred parameters that were statistically significantly different between the left and the right sides of the mesencephalon in patients. All the histogram-based percentiles, which give the highest grey-level value under which a given percentage of the pixels in the image are contained, were statistically significantly different (p < 0.001). Other texture parameters which were most often statistically significantly different consisted mainly of parameters derived from the co-occurrence matrix which gave the highest grey-level value under which a given percentage of the pixels in the image are contained. We observed that there were statistically differing run-length matrix-based parameters, giving information about the spatial variation of gray-level values, between hemispheres in patients but not in healthy controls. This may indicate that the presence of these texture parameters is related to the damage. Clearly there are not so many texture differences between sides in the area of mesencephalon in healthy controls than in MTBI patients.

The ten most discriminative texture features for separation of hemispheres in the area of mesencephalon as identified by calculation of Fisher coefficients, were mainly derived from autoregressive model and the co-occurrence matrix in both patients and controls. Features derived from the autoregressive model; Teta2, Teta3 (p < 0.001) were significantly different between hemispheres in patients and also in controls. Other selected parameters which were statistically different in patients were not different in controls and vice versa. The difference between texture parameters between patients and healthy controls may due to the fact that the injury of the patients has caused complexity in the structure of mesencephalon due to some axonal tearing.

In our study, the texture parameters of WM between hemispheres on different levels were analyzed with Wilcoxon test. Since texture properties are evaluated on a millimetre scale, they may capture the local coherence, direction, and density of fiber bundles, their myelinisation status, the density and direction of vessels supplying and draining WM. According to our study the parameters between WM hemispheres differed most on the level of corona radiata (level 2) in patients. There was not much difference between levels in healthy volunteers. The significantly differing parameters were mainly based on histogram and co-occurrence matrix. And again the run-length matrix-based parameters were statistically different in patients only. It is necessary to take into account that the human brain is asymmetric in structure and function and some of these significant differences in parameters between hemispheres are possibly attibutable to this since less difference was observed on healthy controls it can be assumed that most of the texture changes are caused by the injury.

The ten most discriminative texture features for the separation of hemispheres in the WM as identified by calculation of Fisher coefficients, were mainly derived from the co-occurrence matrix in both patients and controls. Only a few parameters were significantly different between hemispheres in both patients and controls on each level.

Texture parameters of WM between different levels were also analyzed. It was observed that many texture parameters of WM on level 1 were statistically significantly different from parameters on levels 2 and 3, but there were not as many different parameters as between the left and the right hemisphere. Texture parameters between the areas of WM anterior-posterior (front, middle and back) on level 3 were analyzed and it was observed that mostly texture parameters in the posterior region differed from the anterior and the central regions in both hemispheres, which is in line with the fact that many times the trauma is located in the frontal or occipital lobe.

According to our results there are significant differences in texture parameters in the segments of CC and between healthy volunteers and MTBI patients. Our study showed that the texture of the body of CC was different in texture from the rostrum or splenium in patients. The CC is the largest fiber bundle in the human brain connecting two cerebral hemispheres with hundreds of millions of fibers. The fiber composition in the CC has been studied in [44,45] and it has been observed that there are least nerve fibers in the body of CC per unit area and Glial cells occupied more of the body of the CC than of the other segments. The different orientation or densities of the fibers may yield different textures so it could be assumed that the textural changes in the body of CC are caused by the different densities and number of the fibers in different regions of CC. However, since it was observed that in healthy controls the body of CC was not different in texture from the rostrum or splenium, we can presume that the texture differences between the body and other segments of CC in assume may be caused by the injury. Again the ten most discriminative parameters differed and there were only a few wavelet-based features which were significantly different in both groups.

Because our patient group all had normal MRI scans it proved to be very challenging to evaluate the texture changes possibly caused by the injury since we could not categorize the patients according to which part of the head the damage may have occurred in. Also, there are variations in brain structure between individuals making it difficult to detect and classify abnormal structural patterns caused by MTBI and making it difficult to place the ROIs in optimal places. We studied if we could detect differences in textures between the hemispheres in patients and controls. Based on this study the ten most discriminating parameters as identified by calculation of Fisher coefficients on each selected region might only be pertinent to the specific subset of patient tested in this current study. Therefore they are not to be generalized but they however give direction to which type of parameters may be applicable also to other subset of patients. Our results show that there are significant differences in texture parameters in cerebral tissue in the area of mesencephalon and also in the segments of CC and in WM on patients and not so much in healthy controls.

## Conclusions

In conclusion, the study indicates that TA could be used to characterize the changes in cerebral tissue in MTBI patients. This study suggests that texture analysis with a variable set of texture features could in the future serve as an adjuvant diagnostic tool along with traditional MRI and DTI imaging for studying MTBI patients. However, to prove an established role of TA in MTBI further studies are needed, likewise comparison of the texture changes with other possible diagnostic findings.

## Competing interests

The authors declare that they have no competing interests.

## Authors' contributions

SS, PD, JÖ, HE designed and coordinated the whole MTBI project. KKH and LH designed this study and PD, SS and HE participated in its coordination. KKH performed the texture data collection and classification and drafted the manuscript. TL performed statistical analyses. MW performed the neuropsychological assessments. SL performed the neurological examinations and participated in the patient data collection. All authors participated in manuscript modifications, read and approved the final manuscript.

## Pre-publication history

The pre-publication history for this paper can be accessed here:

http://www.biomedcentral.com/1471-2342/10/8/prepub

## Supplementary Material

Additional file 1 **Supplementary table**. Texture parameters used in analysis. Click here for file
