# Supplementary material for: Texture analysis of MR images of patients with Mild Traumatic Brain Injury
Source: BMC Med Imaging. 2010 May 12;10:8. doi: 10.1186/1471-2342-10-8 (PMC3161385; doi:10.1186/1471-2342-10-8)
Supplement: Additional file 1 — Supplementary table. Texture parameters used in analysis. [file 1471-2342-10-8-S1.PDF]

Additional file 1 - Texture parameters used in analysis

Histogram
Mean, variance, skewness, kurtosis, percentiles 1%, 10%, 50%, 90% and 99%

Absolute gradient
Mean, variance, skewness, kurtosis and percentage of pixels with nonzero Gradient
(information about the spatial variation of gray-level values)

Co-occurrence matrix (COM)
Angular second moment, contrast, correlation, sum of squares, inverse difference moment, sum average, sum variance, sum entropy, entropy, difference variance and difference entropy
(information about the gray-level value distribution of pairs of pixels, separated by a defined distance in a given direction),

Run-length matrix (RLM)
Run-length nonuniformity, grey-level nonuniformity, long-run emphasis, shortrun emphasis and fraction of image in runs
(information about runs of pixels with the same gray-level values in a given direction)

Autoregressive model (ARM)
Theta: model parameter vector, 4 parameters; Sigma: standard deviation of the driving noise
(description of texture based on the statistical correlation between neighbouring pixels)

Wavelet
Energy of the wavelet coefficients in subbands
